# Supplementary material for: Manipulating Reaction Energy Coordinate Landscape of Mechanochemical Diaza-Cope Rearrangement
Source: Molecules. 2022 Apr 15;27(8):2570. doi: 10.3390/molecules27082570 (PMC9027841; doi:10.3390/molecules27082570)
Supplement: Supplementary file 1 [file molecules-27-02570-s001.zip › molecules-1681665-supplementary.pdf]

molecules

Supporting Information

Manipulating Reaction Energy Coordinate Landscape of Mechanochemical Diaza-Cope Rearrangement.

Tingting Cheng [1,†], Wenxian Ma [1,2,3,†], Hao Luo [1,†], Yangzhi Ye [1] and KaKing Yan [1,*]

[1] School of Physical Science and Technology, ShanghaiTech University, Shanghai 201210, China
[2] Shanghai Advanced Research Institute, Chinese Academy of Sciences, Shanghai 201203, China
[3] University of Chinese Academy of Sciences, Beijing 100049, China

Table of Contents

1. Ball milling equipment (Figure S1)
2. Silica gel loading effect on mechanochemical DCR reaction between (R,R)-**1** and **2a**. (Table S1)
3. Monitoring mechanochemical reaction with **1** and **2a with** silica gel (Figure S2-S3)
4. Monitoring mechanochemical reaction with **1** and **2a without** silica gel (Figure S4-S5)
5. FT-IR spectra of untreated silica gel, **SG**$_{(314)}$ and **SG**$_{(528)}$ (Figure S6)
6. PXRD studies (Figure S7)
7. General procedure for mechanochemical diaza-Cope rearrangement (Figure S8-S20)

1. Ball milling equipment

Ball-milling reactions were performed with a Shanghai Jing Xin JXFSTPRP-24 mixer mill. Polypropylene (PP) milling tubes (2 ml), stainless steel milling vessels (2 ml (JX-KG0177)), stainless steel milling vessels (15 ml (JX-KG0180)), and grinding balls (3.0 mm (JX-GZ0137) (stainless-steel), (JX-YG0123) (ZrO_2) and 12.0 mm (JX-GZ0142)) were purchased from Shanghai Jing Xin.

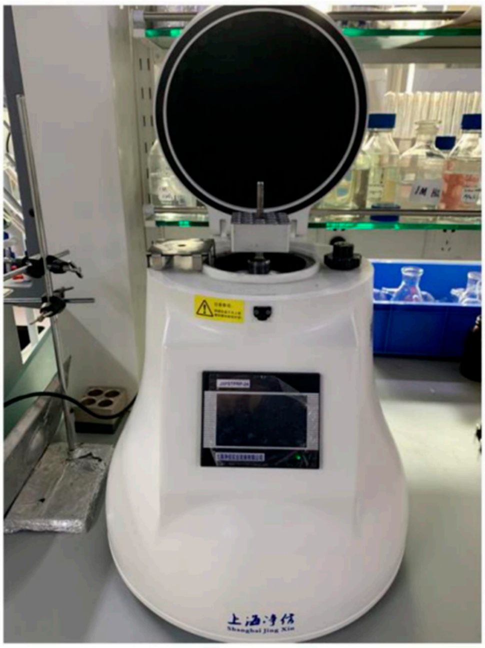

Shanghai Jing Xin JXFSTPRP-24 mixer mill

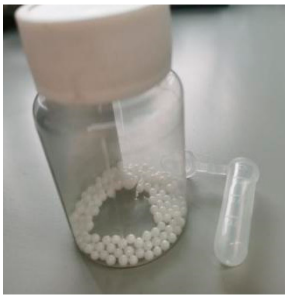

3.0 mm ZrO_2 grinding balls and 2 ml PP milling tube

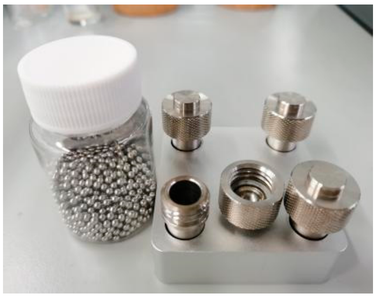

12.0 mm stainless-steel grinding balls and 15 ml stainless-steel milling vessel

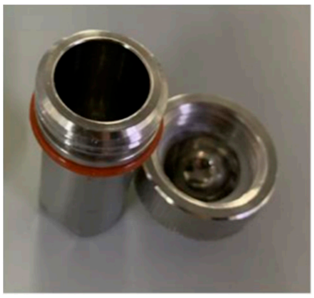

3.0 mm stainless-steel grinding ball and 2 ml stainless-steel milling vessel

Figure S1. (Top) Ball-milling machine, and (bottom) ball-milling vessels and milling balls used in this study.

Silica gel loading effect on mechanochemical DCR reaction between (*R*,*R*)-**1** and **2a**.

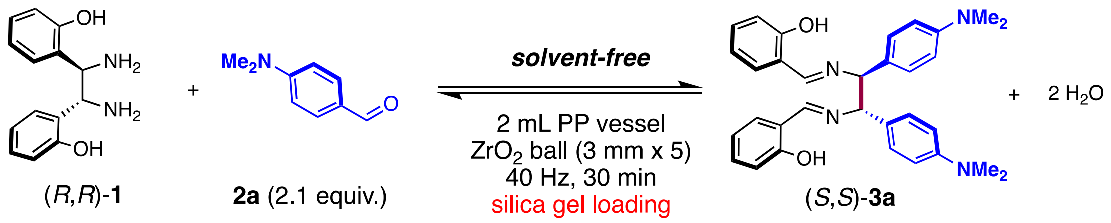

Entry	mass of silica gel (mg)[b]	NMR yield (%)[a]
1	0	14
2	10	48
3	20	59
4	30	62
5	50	75
6	100	76

Table S1. Silica gel loading effect on mechanochemical DCR reaction between (*R*,*R*)-**1** and **2a**. [a] NMR (^{1}H) spectrum is taken immediately after ball-milling reaction at the reported time. Yield is determined by comparing the ^{1}H NMR integration of **3a** to that of an internal standard (dibromomethane) with known concentration. [b] 300-400 mesh.

Monitoring mechanochemical reaction with **1** (0.082 mmol) and **2a** (0.174 mmol) **with** silica gel

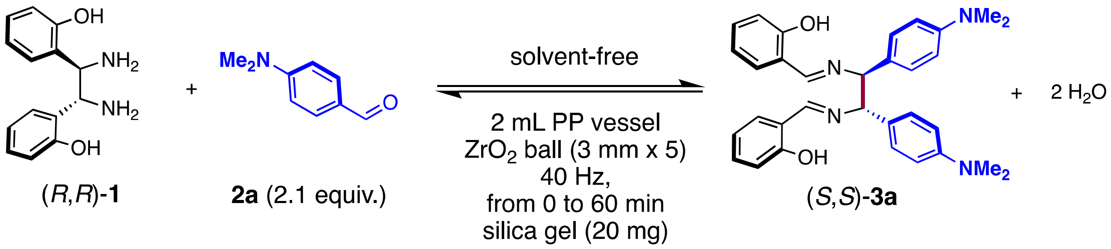

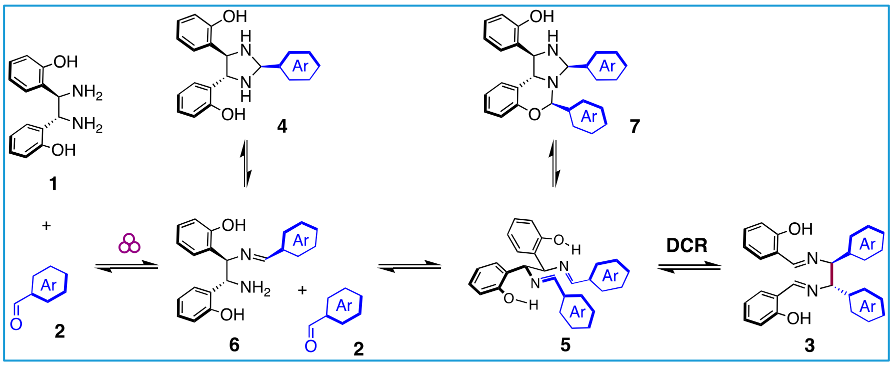

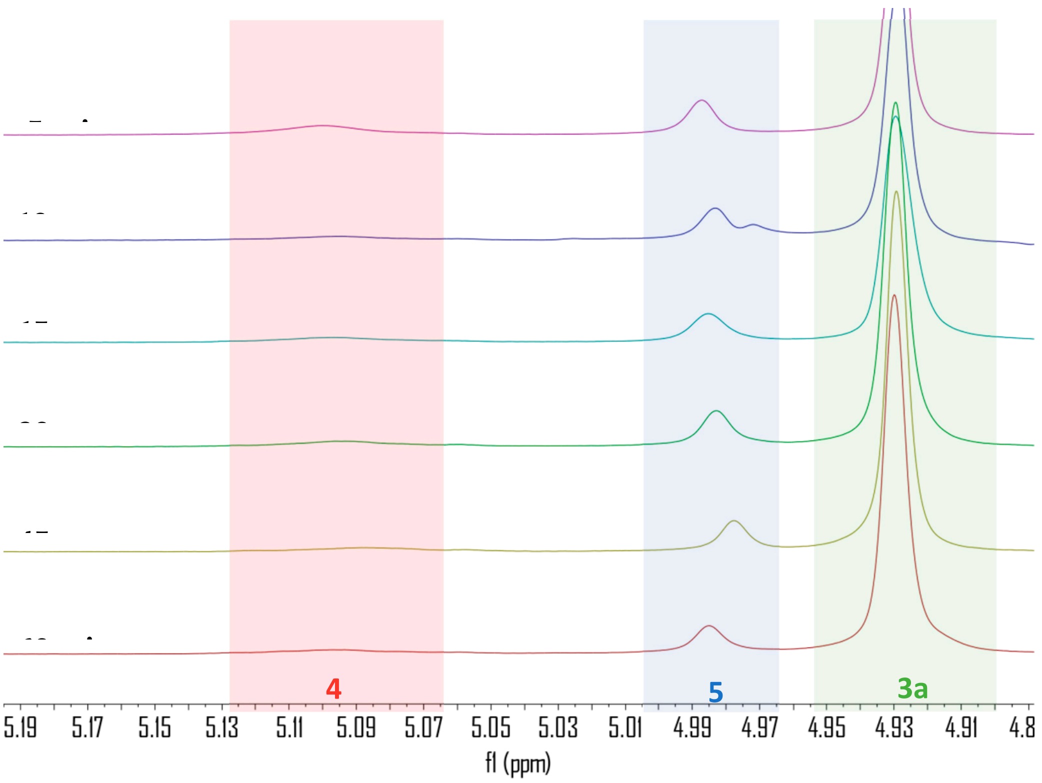

Figure S2. ^{1}H NMR spectra of reaction time course monitoring **with** silica gel additive. The milled sample was dissolved in DMSO-d_6 and the NMR sample was injected into an NMR spectrometer as quickly as possible (within 2 min) to minimize further solution reaction of the milled solid sample. After that, data acquisition began immediately to acquire the ^{1}H NMR spectrum. Ratios of different species are determined by NMR integrations against an internal standard with known concentration.

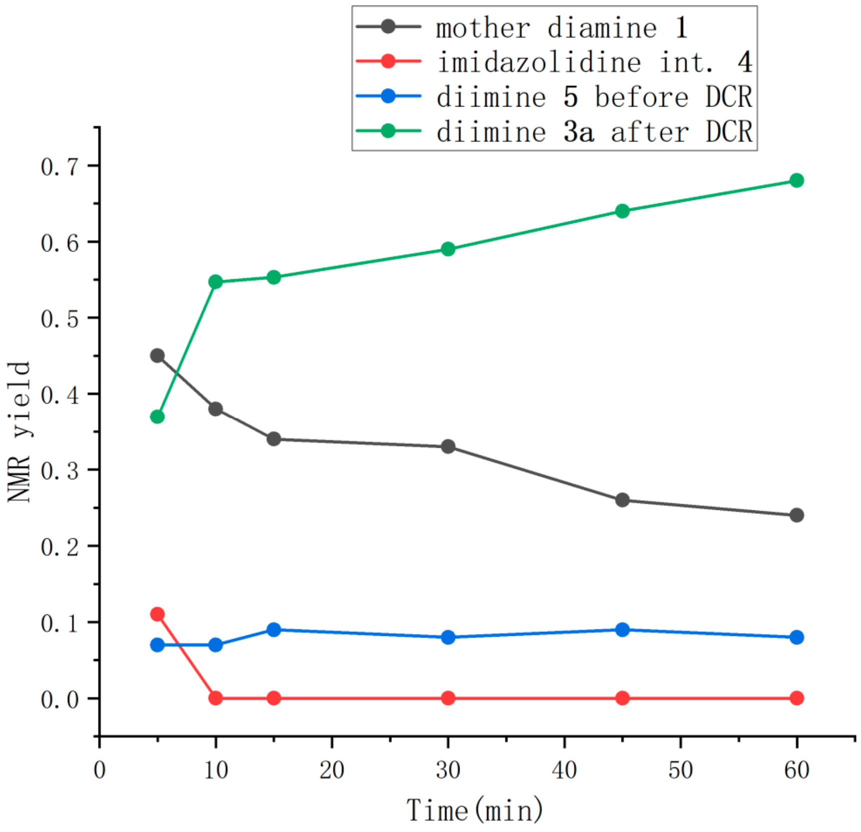

Figure S3. Plot of reaction time vs. NMR yield of different species in mechanochemical DCR **with** silica gel: **1** (mother diamine), **4** (intermediate), **5** (diimine intermediate before DCR) and **3a** (DCR product).

Monitoring mechanochemical reaction with **1** (0.082 mmol) and **2a** (0.174 mmol) **without** silica gel

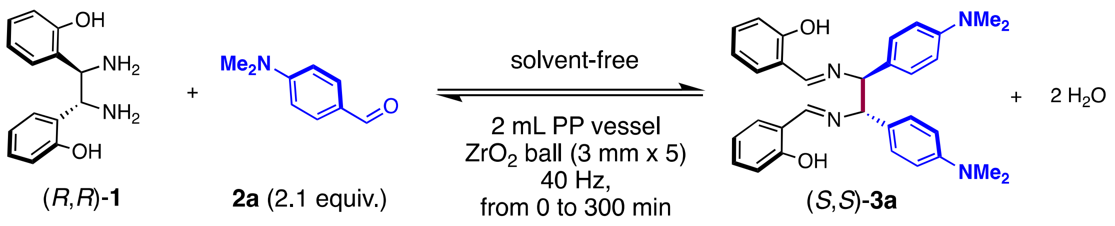

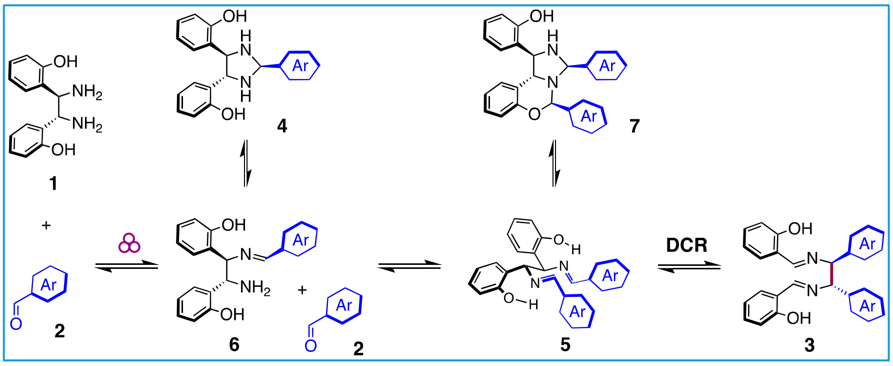

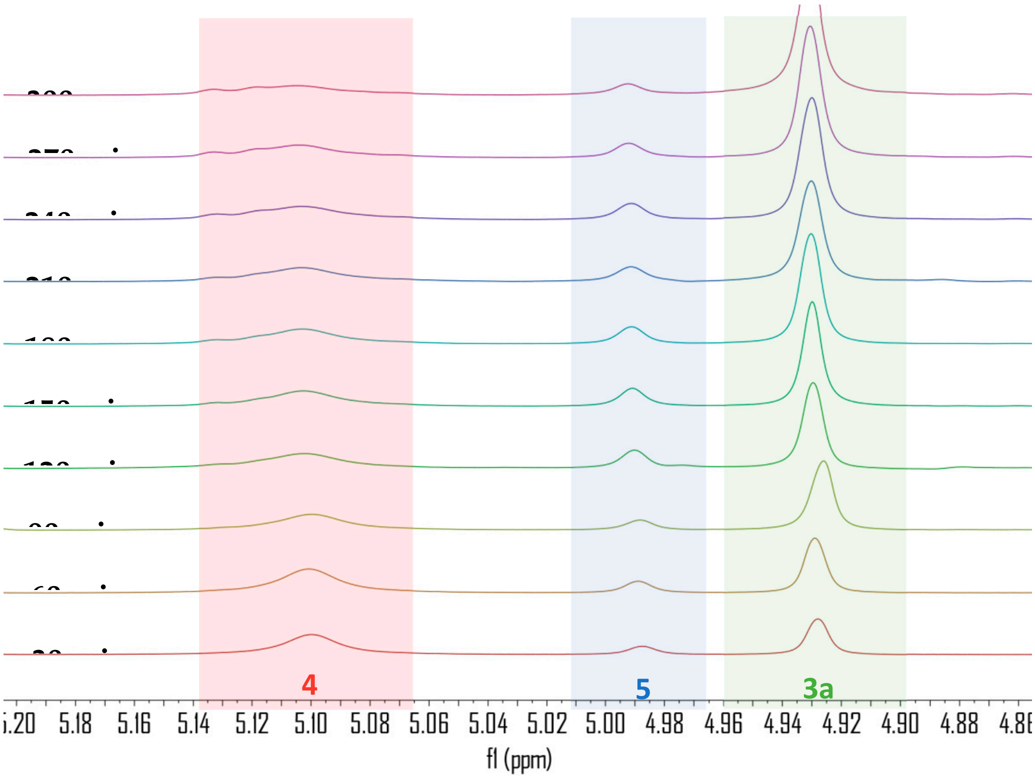

Figure S4. ^{1}H NMR spectra of reaction time course monitoring **without** silica gel. The milled sample was dissolved in DMSO-d_6 and the NMR sample was injected into an NMR spectrometer as quickly as possible (within 2 min) to minimize further solution reaction of the milled solid sample. After that, data acquisition began immediately to acquire the ^{1}H NMR spectrum. Ratios of different species are determined by NMR integrations against an internal standard with known concentration.

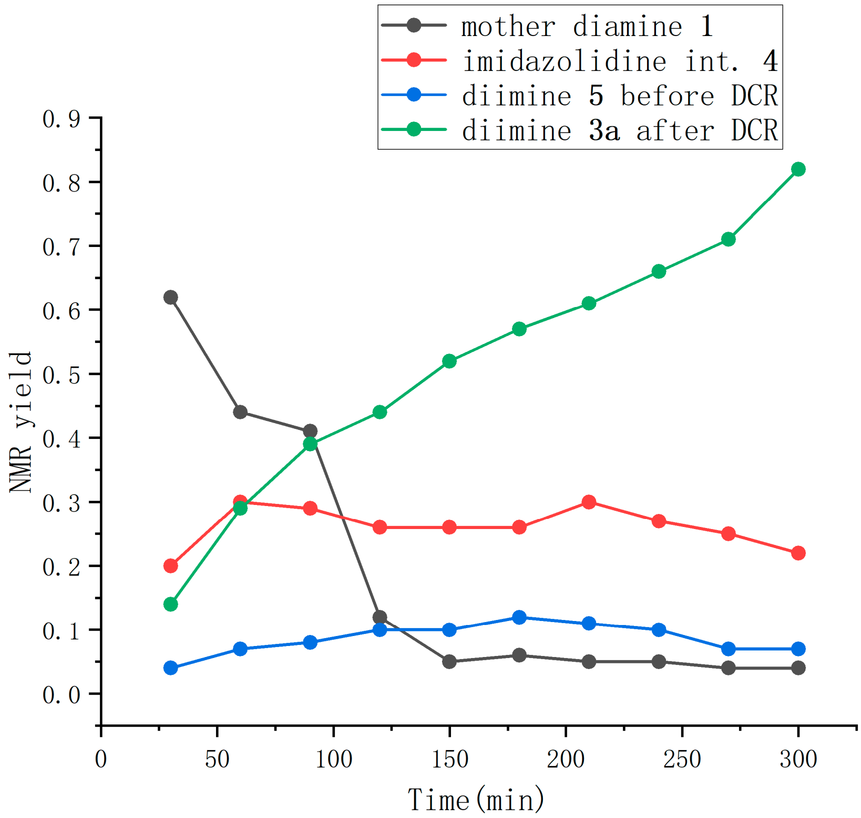

Figure S5. Plot of reaction time vs. NMR yield of different species in mechanochemical DCR **without** silica gel: **1** (mother diamine), **4** (intermediate), **5** (diimine intermediate before DCR) and **3a** (DCR product).

FT-IR spectra of $\mathbf{SG}_{(314)}$ and $\mathbf{SG}_{(528)}$

- Activated silica gel samples were thermolyzed under dynamic vacuum at the designated temperature with a heating furnace for 5 h, prior to mechanochemical diaza-Cope rearrangement.

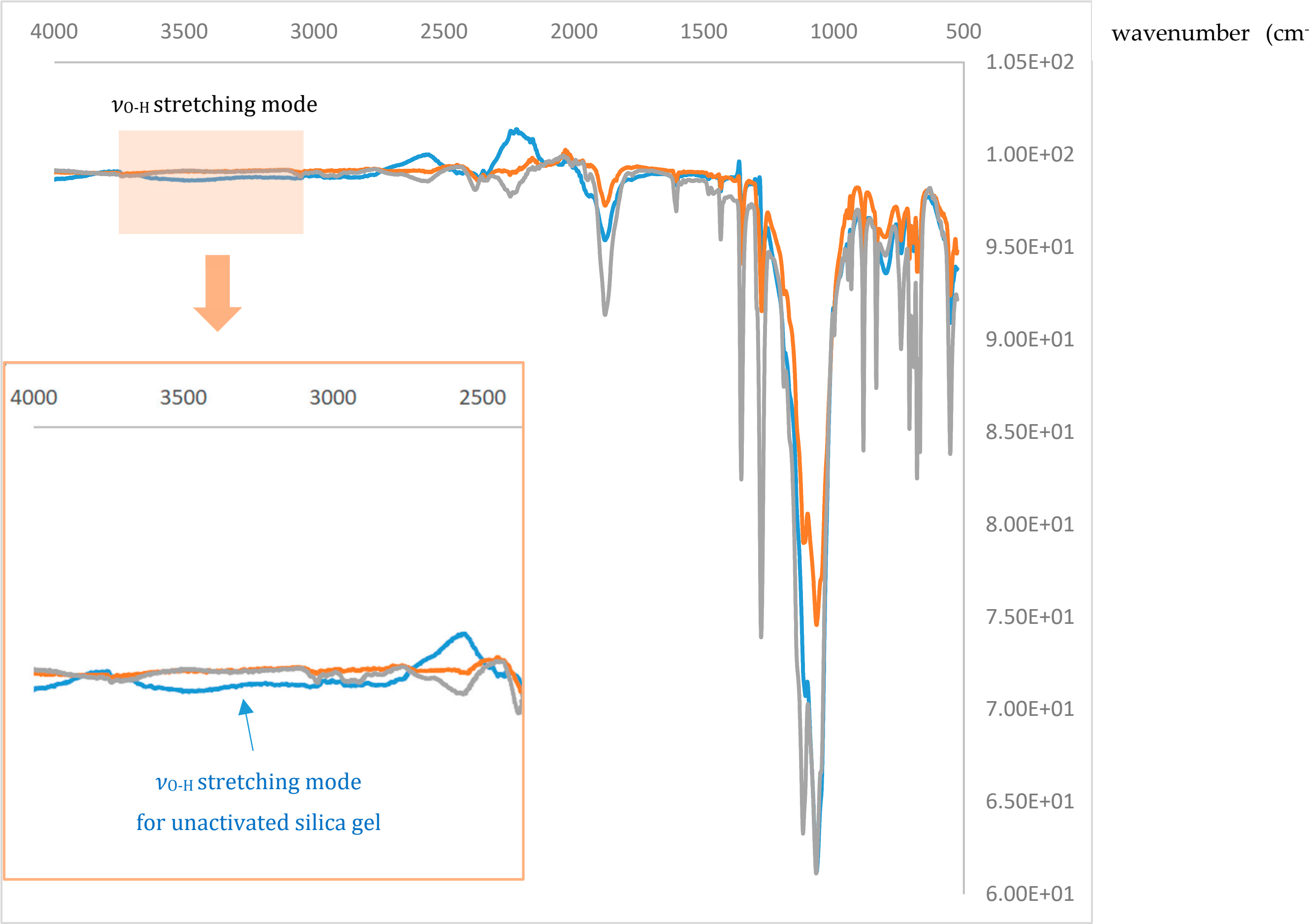

Figure S6. FT-IR spectra of silica gel$_{(314)}$ and silica gel$_{(528)}$.

Blue: silica gel
Orange: silica gel$_{(528)}$
Grey: silica gel$_{(314)}$

From ~2800-3500 cm^{-1}, untreated silica gel shows a broad peak which belongs to -OH stretching mode, while **SG**$_{314}$ and **SG**$_{528}$ both have no signals. This confirms the condensation of silanol groups under thermal treatment.

1. PXRD studies

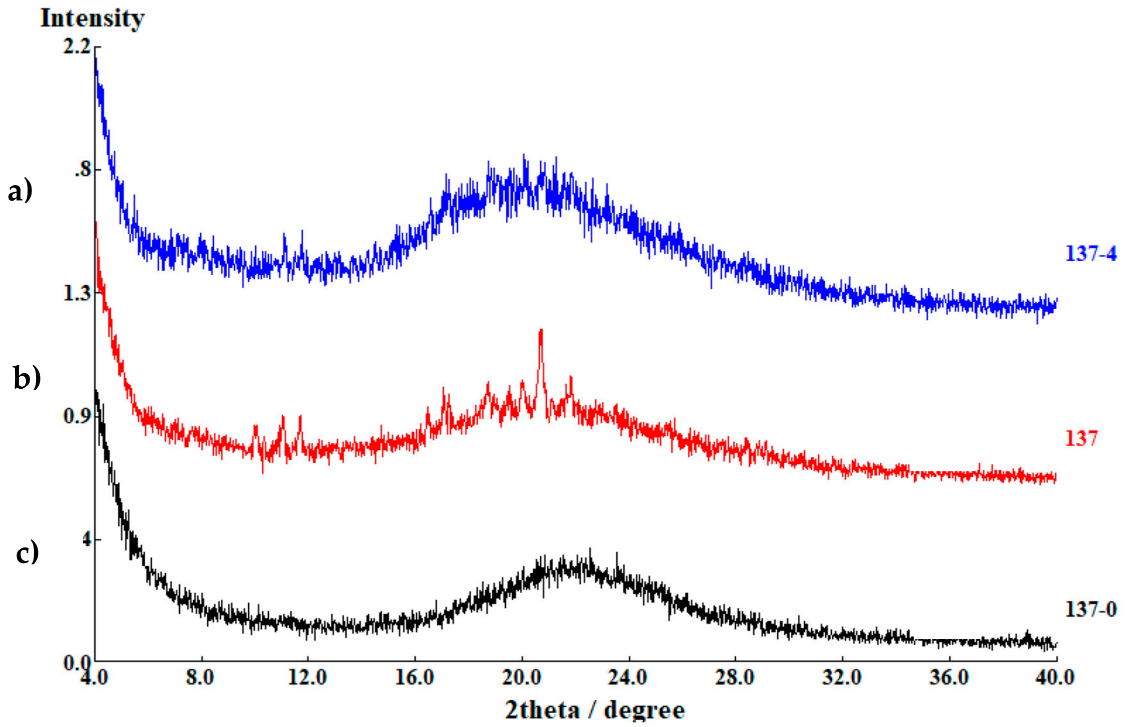

Figure S7. PXRD patterns of a) **1** (0.041 mmol) and **2a** (0.087 mmol) under 5 min, 40 Hz ball milling containing silica gel and 7 days aging without any purification; b) **1** (0.041 mmol) and **2a** (0.087 mmol) under 5 min, 40 Hz ball milling containing silica gel without any purification; c) silica gel after 5 min, 40 Hz ball milling.

- The PXRD spectra showed mainly the amorphous phase from silica gel with a broad peak from 15° to 28°.

2. General procedure for mechanochemical diaza-Cope rearrangement (0.2 mmol scale)

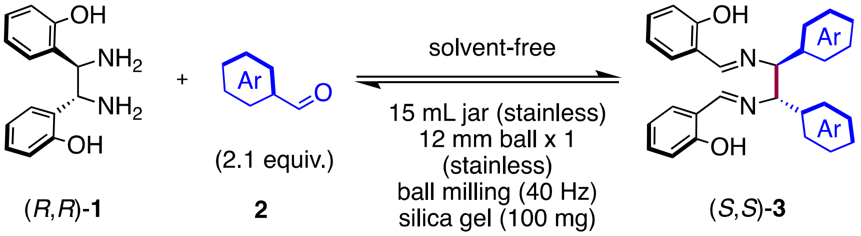

(1*R*,2*R*)-1,2-Bis(2-hydroxyphenyl)ethylenediamine **1**, arylaldehyde **2** (2.1 equiv.) were placed in a stainless steel ball-milling vessel (15 mL) loaded with one grinding ball (stainless steel, diameter: 12 mm). After the vessel was closed in open air, the vessel was placed in the ball mill machine. After 4 h of ball-milling at 40 Hz, with a periodic 5 min pause for every 1 h of milling, the solid residual was dissolved in DCM, and then purified by chromatography (eluent hexane: ethyl acetate = 10 :1 to 1:1) to give product **3**.

2,2'-[[(1*S*,2*S*)-1,2-di(2-iodophenyl)-1,2-ethanediyl]bis[(*E*)-nitrilomethylidyne]]bi-sphenol (**3e**).

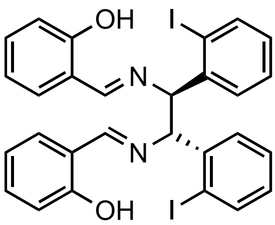

Prepared according to general procedure from **1** (50 mg, 0.20 mmol) and 2-iodobenzaldehyde (**2e**) (98 mg, 0.42 mmol) to give the title compound as a yellow solid (82 mg, 61%).

^{1}H NMR ($CDCl_3$, 400 MHz, 25 °C) δ 13.06 (s, 2H), 8.44 (s, 2H), 7.69 (ddd, *J* = 15.6, 7.9, 1.5 Hz, 4H), 7.33-7.26 (m, 4H), 7.19 (dd, *J* = 7.7, 1.7 Hz, 2H), 6.94-6.91 (m, 2H), 6.90-6.85 (m, 2H), 6.82 (td, *J* = 7.4, 1.1 Hz, 2H), 5.44 (s, 2H).

^{13}C{^{1}H} NMR ($CDCl_3$, 101 MHz, 25 °C) δ 166.73, 160.90, 140.61, 139.68, 132.82, 132.05, 130.68, 129.64, 128.67, 118.89, 118.53, 116.93, 100.52, 80.33.

HRMS (ESI) calculated for $C_{28}H_{22}I_2N_2O_2$, $[M+H]^+$: 672.9843, Found: 672.9843.

HPLC: Chiralpak IC column, 95:5 n-hexane/ iso-propanol, 1 ml/min; (*R*,*R*)-**3e** t_R = 6.0 min, (*S*,*S*)-**3e** t_R = 7.0 min for racemic sample of **3e**.

ee: 98%.

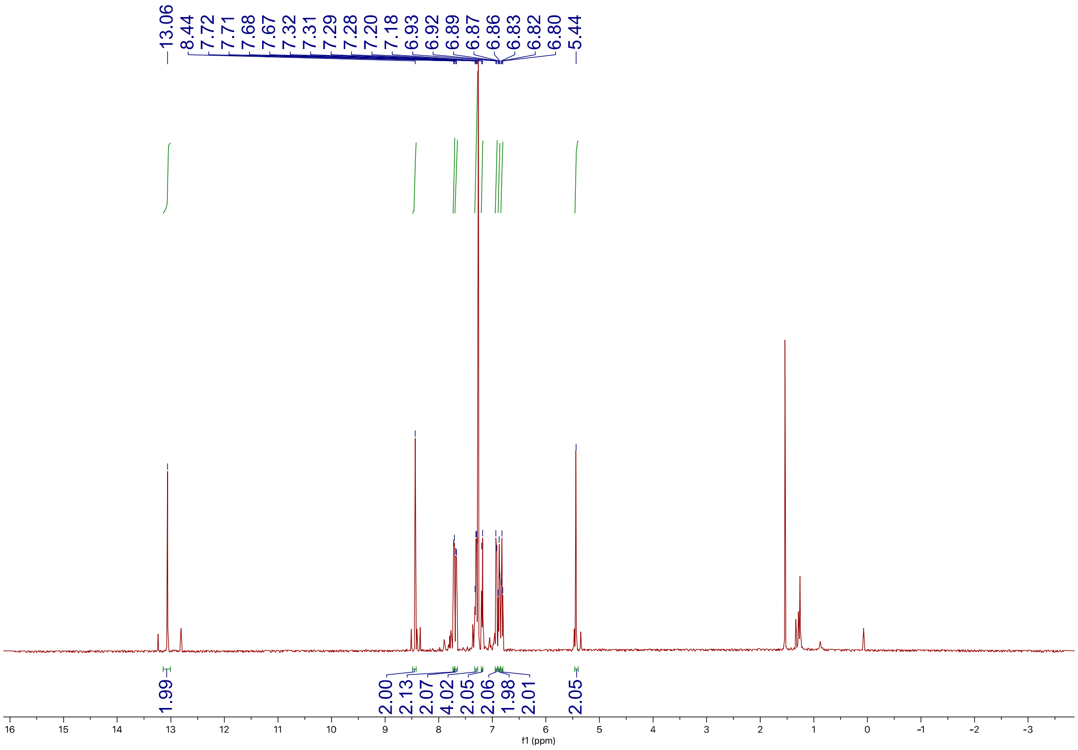

Figure S8. ^{1}H NMR spectrum of **3e**.

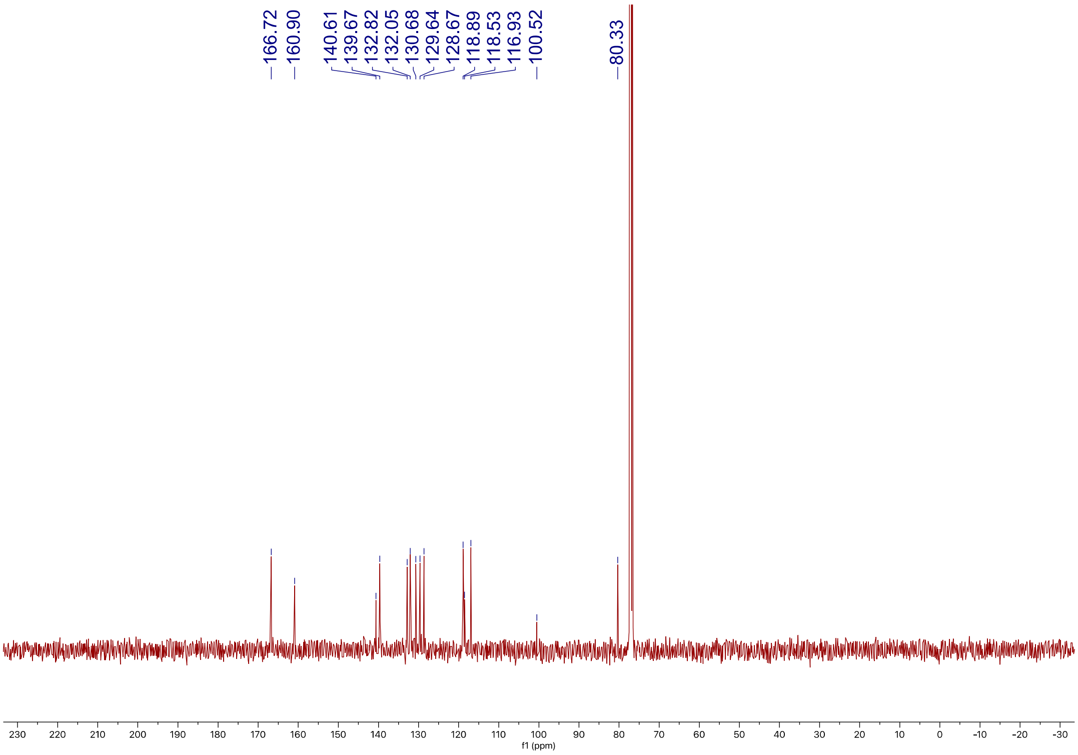

Figure S9. $^{13}C\{^{1}H\}$ NMR spectrum of **3e**.

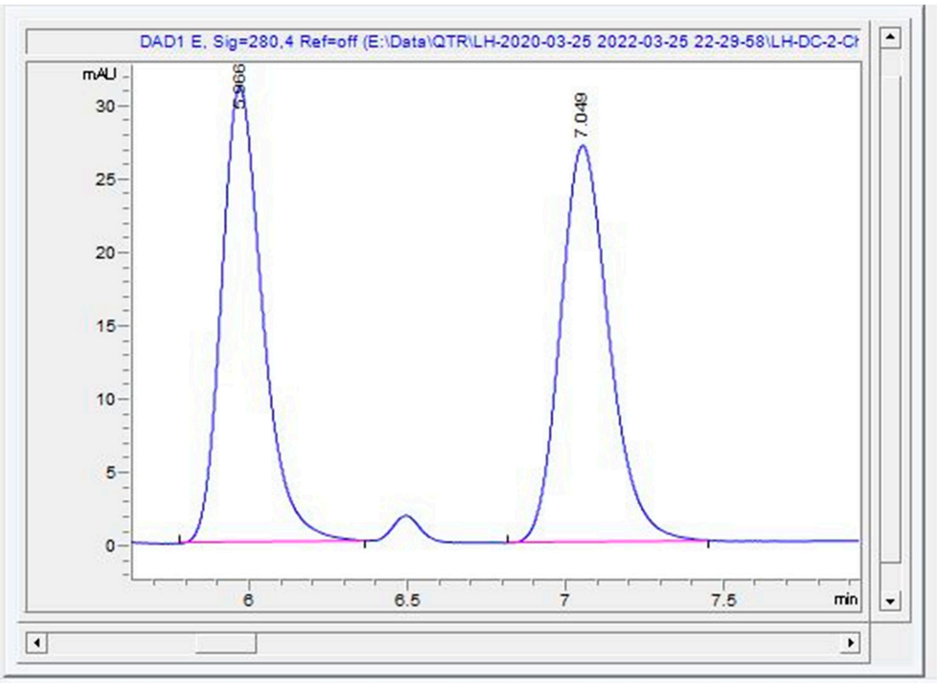

#	Time	Type	Area	Height	Width	Area%	Symmetry
1	5.966	BB	280.3	30.9	0.1357	49.296	0.746
2	7.049	BB	288.3	26.9	0.1569	50.704	0.804

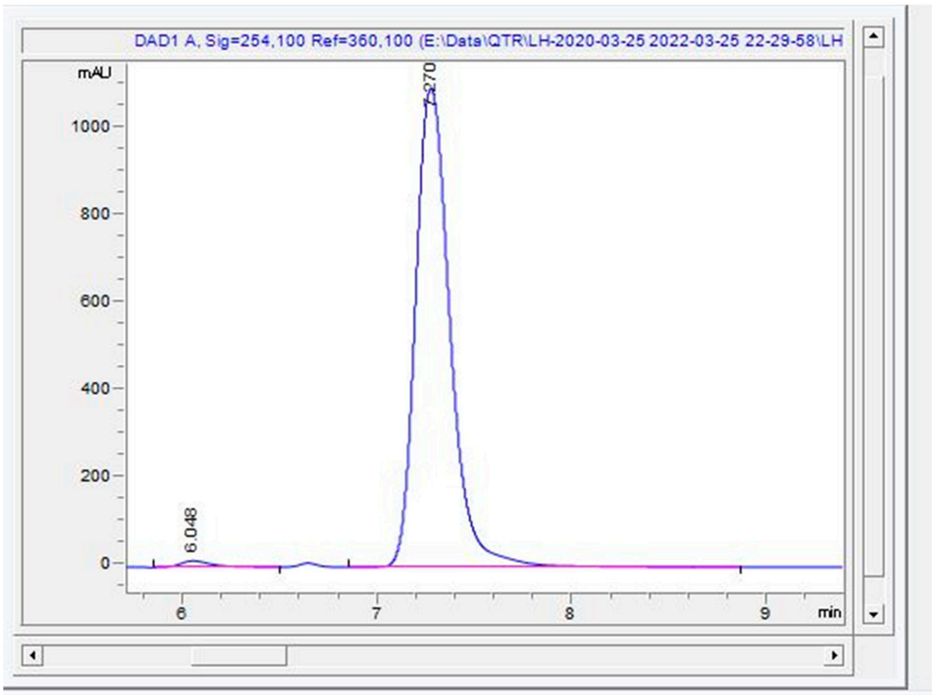

#	Time	Type	Area	Height	Width	Area%	Symmetry
1	6.048	BB	162.2	14.6	0.1619	1.191	0.651
2	7.27	BB	13463.1	1095.2	0.1889	98.809	0.738

Figure S10. HPLC analysis of (top) racemic sample and (bottom) (*S*,*S*) sample of **3e**.

2,2'-[[(1*S*,2*S*)-1,2-di((2-phenyl)phenyl)-1,2-ethanediyl]bis[(*E*)-nitrilomethylidyne]]bi-sphenol (**3f**)

Prepared according to general procedure from **1** (50 mg, 0.20 mmol) and [1,1'-bi-phenyl]-2-carbaldehyde (**2f**) (78 mg, 0.42 mmol) to give the title compound as a yellow solid (78 mg, 68%).

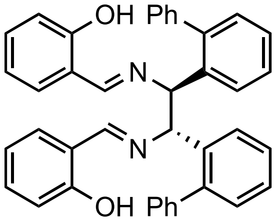

^{1}H NMR ($CDCl_3$, 400 MHz, 25 °C) δ 13.05 (s, 2H), 8.00 (s, 2H), 7.23 (dt, *J* = 7.1, 4.0 Hz, 4H), 7.11-6.94 (m, 12H), 6.88-6.84 (m, 2H), 6.79-6.68 (m, 6H), 6.62 (td, *J* = 7.5, 1.1 Hz, 2H), 4.87 (s, 2H).

^{13}C{^{1}H} NMR ($CDCl_3$, 101 MHz, 25 °C) δ 165.64, 160.82, 142.08, 140.68, 135.95, 132.49, 131.73, 129.93, 129.66, 128.67, 128.14, 128.03, 127.83, 127.29, 127.18, 127.00, 118.75, 118.56, 116.98, 116.77.

HRMS (ESI) calculated for $C_{40}H_{32}N_2O_2$, $[M+H]^+$: 573.2537, Found: 573.2544.

HPLC: Chiralpak IA column, 98:2 n-hexane/iso-propanol, 1 ml/min; (*R*,*R*)-**3f** t_R = 14.3 min, (*S*,*S*)-**3f** t_R = 4.7 min for racemic sample of **3f**.

ee: 96%.

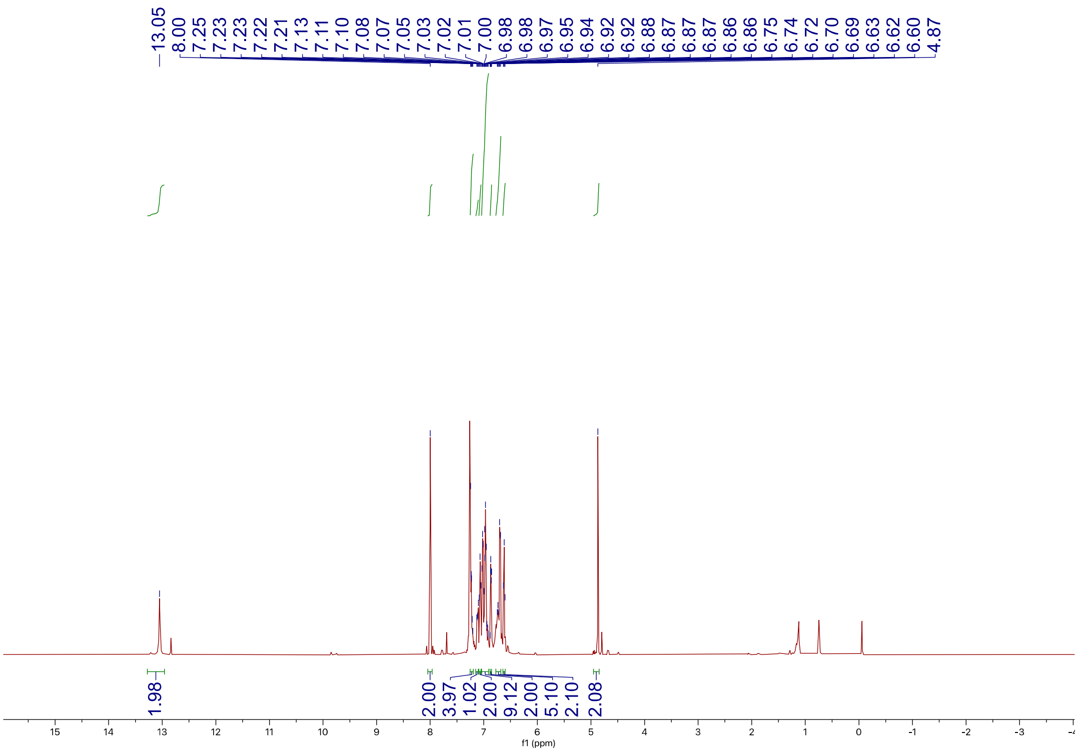

Figure S11. ^{1}H NMR spectrum of **3f**.

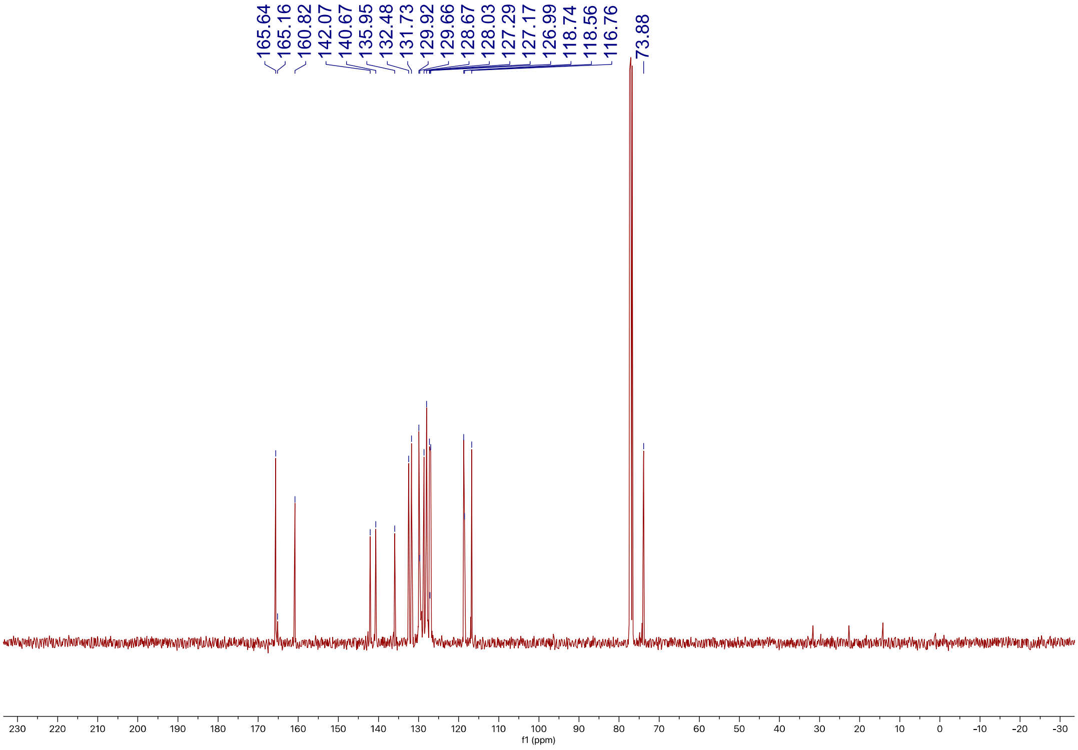

Figure S12. ^{13}C{^{1}H} NMR spectrum of **3f**.

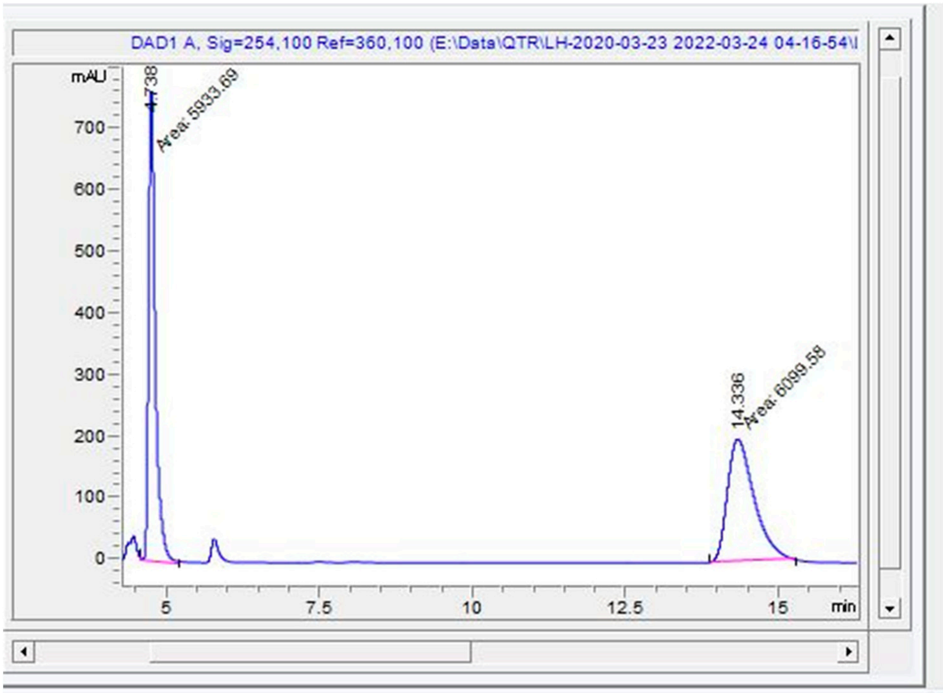

#	Time	Type	Area	Height	Width	Area%	Symmetry
1	4.738	MM	5933.7	766.2	0.1291	49.311	0.588
2	14.336	MM	6099.6	199.2	0.5102	50.689	0.603

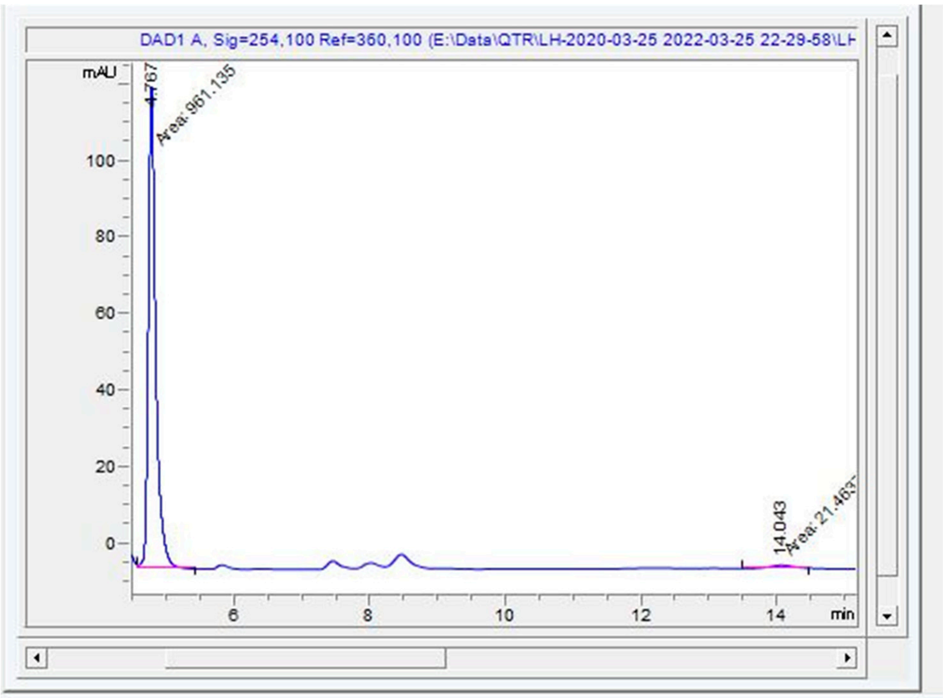

#	Time	Type	Area	Height	Width	Area%	Symmetry
1	4.767	FM	961.1	125.8	0.1273	97.816	0.666
2	14.043	MM	21.5	8.6E-1	0.4144	2.184	1.15

Figure S13. HPLC analysis of (top) racemic sample and (bottom) (*S*,*S*) sample of **3f**.

2,2'-[[(1*S*,2*S*)-1,2-di(2-(4,4,5,5-tetramethyl-1,3,2-dioxaborolan-2-yl)phenyl)-1,2-ethanediyl]bis[(*E*)-nitrilomethylidyne]]bisphenol (**3h**).

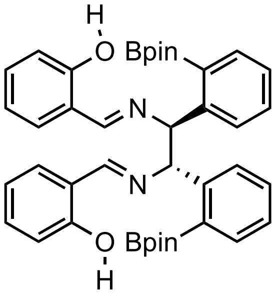

Prepared according to general procedure from **1** (50 mg, 0.20 mmol) and 2-(4,4,5,5-tetramethyl-1,3,2-dioxaborolan-2-yl)benzaldehyde (**2h**) (98 mg, 0.42 mmol) to give the title compound as a yellow solid (30 mg, 22%).

^{1}H NMR ($CDCl_3$, 400 MHz, 25 °C) δ 13.67 (s, 2H), 8.52 (s, 2H), 7.75 (d, *J* = 7.8 Hz, 2H), 7.58 (dd, *J* = 7.5, 1.5 Hz, 2H), 7.35 – 7.29 (m, 2H), 7.25 – 7.21 (m, 2H), 7.17 (dd, *J* = 7.7, 1.7 Hz, 2H), 7.06 (td, *J* = 7.4, 1.1 Hz, 2H), 6.87 (d, *J* = 8.2 Hz, 2H), 6.79 (td, *J* = 7.4, 1.1 Hz, 2H), 6.11 (s, 2H), 1.44 (s, 12H), 1.38 (s, 12H).

^{13}C NMR ($CDCl_3$, 101 MHz, 25 °C) δ 165.89, 161.07, 145.97, 136.22, 132.15, 131.69, 131.15, 128.14, 126.49, 119.02, 118.52, 116.71, 83.81, 75.00, 25.05.

^{11}B NMR ($CDCl_3$, 128 MHz, 25 °C) δ 4.30.

HRMS (ESI) calculated for $C_{40}H_{46}B_2N_2O_6$, [M+H]$^+$: 673.3615, Found: 673.3659.

HPLC: Chiralpak IC column, 95:5 n-hexane/iso-propanol, 1 ml/min; (*R*,*R*)-**3h** t_R = 5.3 min, (*S*,*S*)-**3h** t_R = 4.4 min for racemic sample of **3h**.

ee: 99%.

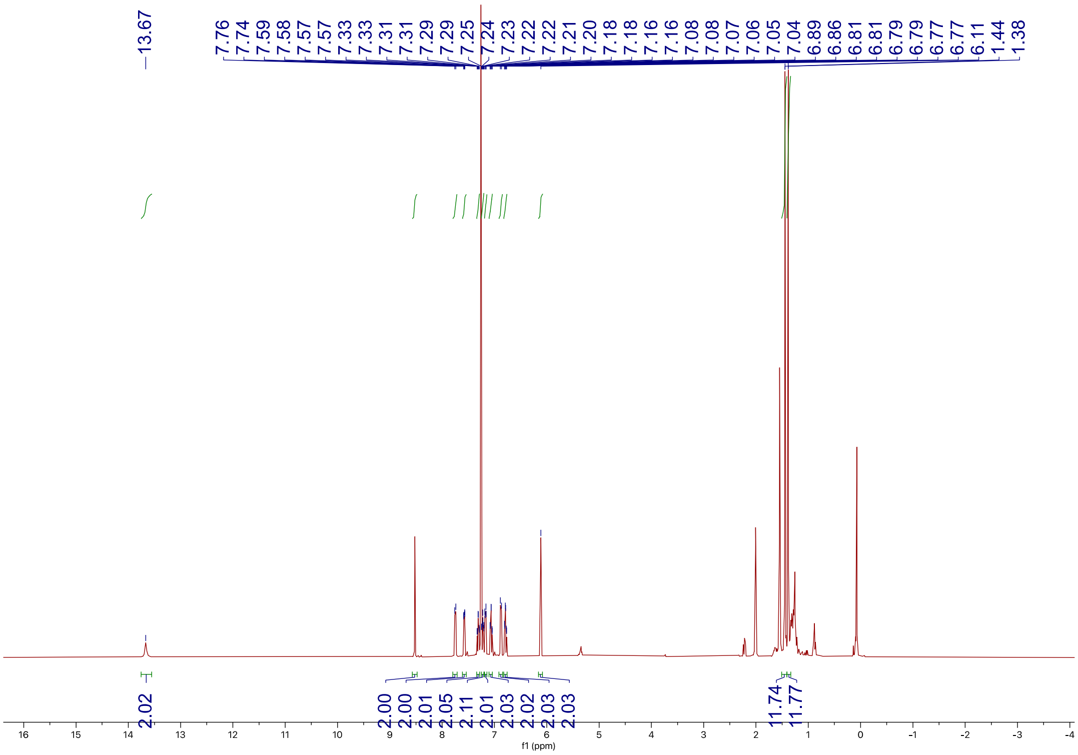

Figure S14. ^{1}H NMR spectrum of **3h**.

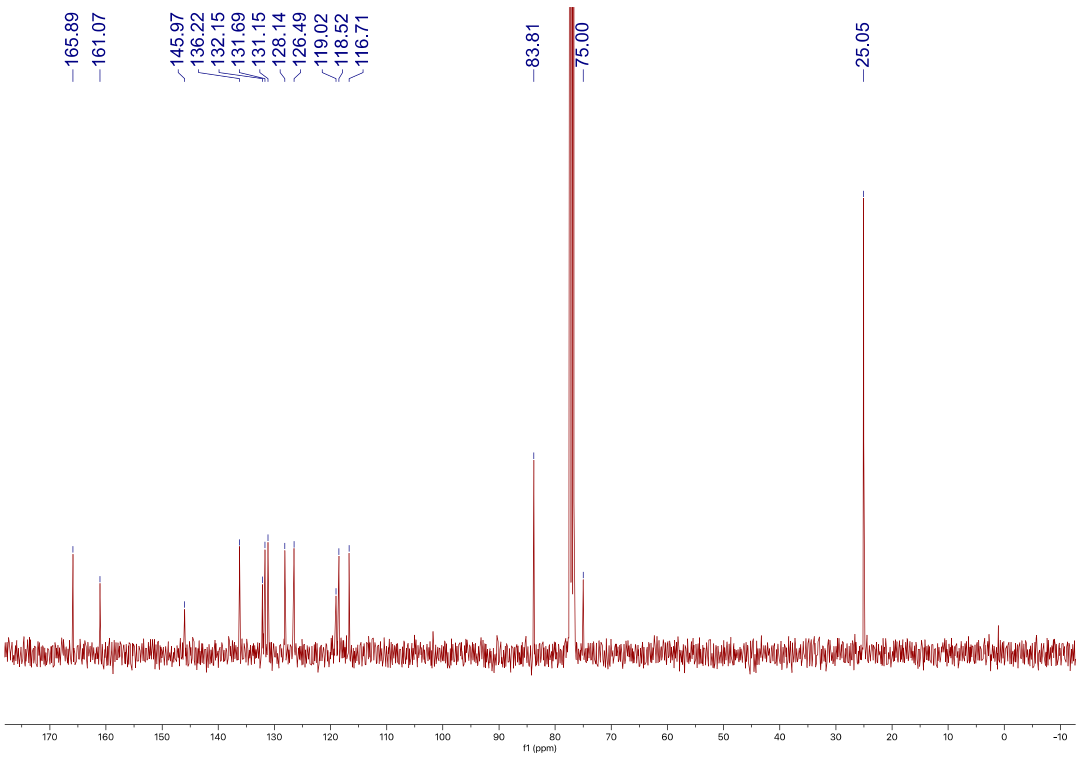

Figure S15. $^{13}C\{^{1}H\}$ NMR spectrum of **3h**.

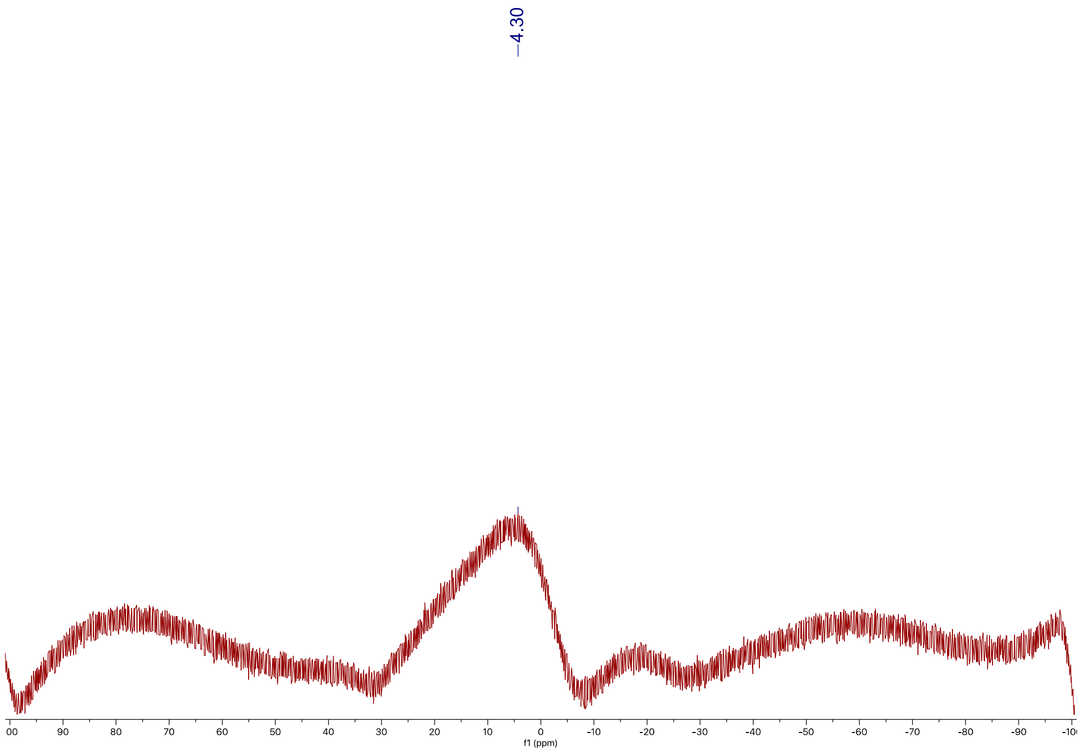

Figure S16. ^{11}B NMR spectrum of **3h**.

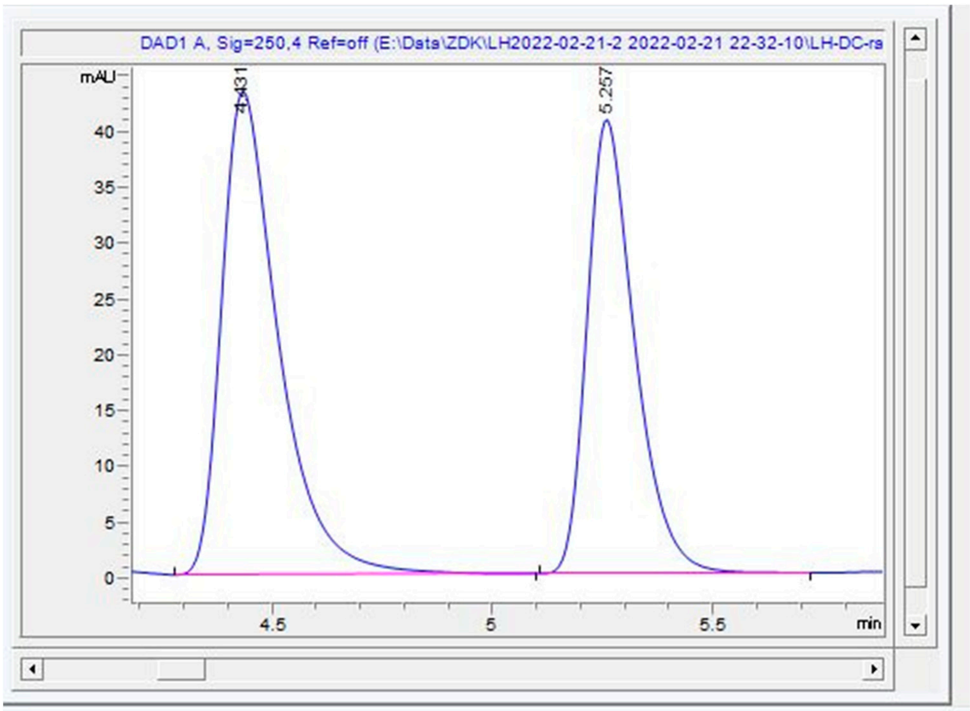

#	Time	Type	Area	Height	Width	Area%	Symmetry
1	4.431	BB	388.8	43.2	0.1349	55.939	0.552
2	5.257	BB	306.2	40.6	0.114	44.061	0.643

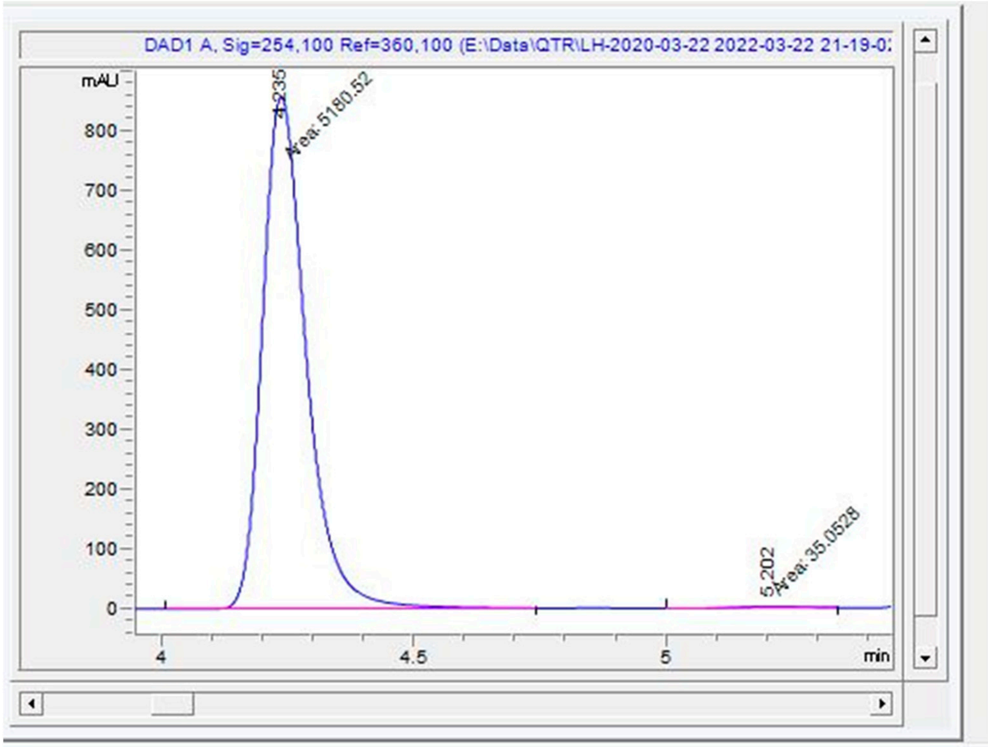

#	Time	Type	Area	Height	Width	Area%	Symmetry
1	4.235	MF	5180.5	857.6	0.1007	99.328	0.715
2	5.202	MM	35.1	3.4	0.1738	0.672	1.077

Figure S17. HPLC analysis of (top) racemic sample and (bottom) (*S*,*S*) sample of **3h**.2,2'-[[(1*S*, 2*S*)-1,2-di(3-bromonaphthalen-2-yl)-1,2-ethanediyl]bis[(*E*)-nitrilomethylidyne]] bisphenol (**3i**)

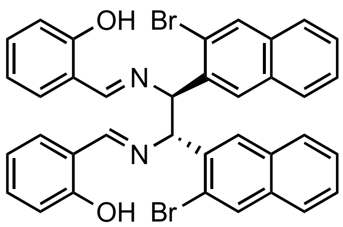

Prepared according to general procedure from **1** (50 mg, 0.20 mmol) and 3-bromo-2-naphthaldehyde (**2i**) (99 mg, 0.42 mmol) to give the title compound as a yellow solid (76 mg, 48%).

^{1}H NMR ($CDCl_3$, 500 MHz, 25 °C) δ 13.28 (s, 2H), 8.42 (s, 2H), 8.26 (d, *J* = 8.6 Hz, 2H), 7.82 (d, *J* = 8.6 Hz, 2H), 7.70 (t, *J* = 8.1 Hz, 4H), 7.52 (t, *J* = 7.7 Hz, 2H), 7.45 (t, *J* = 7.5 Hz, 2H), 7.28 (t, *J* = 7.6 Hz, 2H), 7.13 (d, *J* = 7.6 Hz, 2H), 6.97 (d, *J* = 8.3 Hz, 2H), 6.80 (t, *J* = 7.5 Hz, 2H), 6.06 (s, 2H).

^{13}C NMR ($CDCl_3$, 101 MHz, 25 °C) δ 167.30, 161.03, 136.03, 133.98, 132.84, 132.13, 132.03, 128.15, 128.05, 128.00, 127.49, 126.85, 123.95, 118.84, 118.58, 117.02, 76.46.

HRMS (ESI) calculated for $C_{36}H_{26}Br_2N_2O_2$, $[M+H]^+$: 679.0413, Found: 679.0427.

HPLC: Chiralpak IC column, 95:5 n-hexane/iso-propanol, 1 ml/min; (*R*,*R*)-**3i** t_R = 7.8 min, (*S*,*S*)-**3i** t_R = 8.8 min for racemic sample of **3i**.   ee>99%

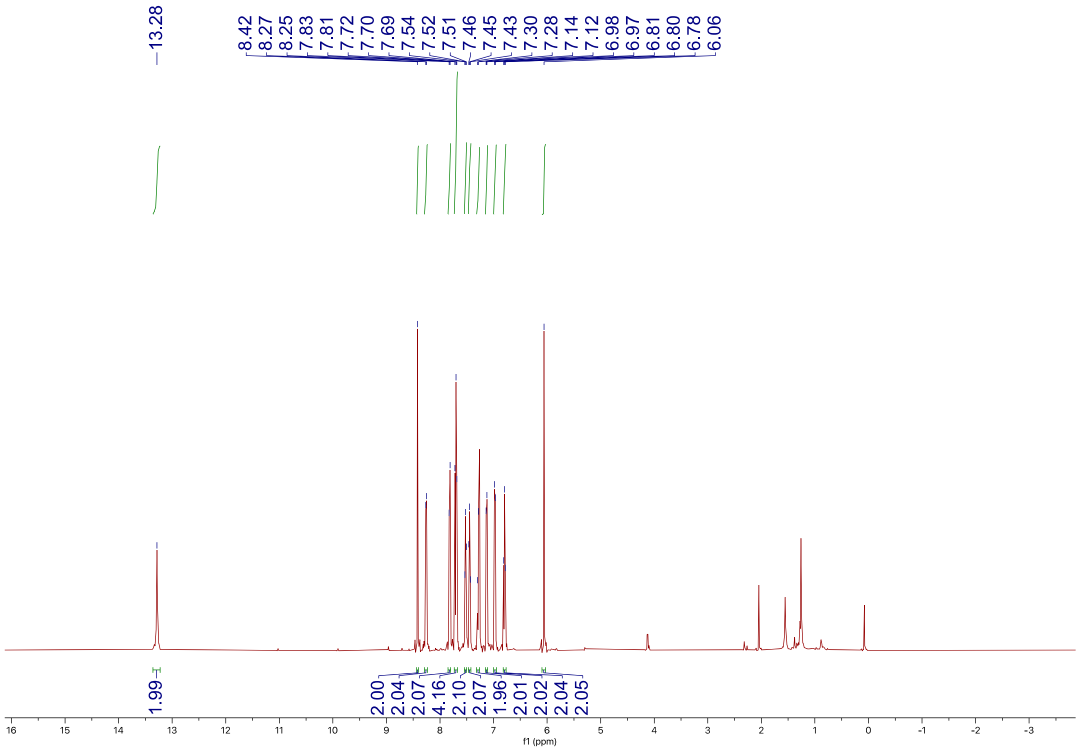

Figure S18. ^{1}H NMR spectrum of **3i**.

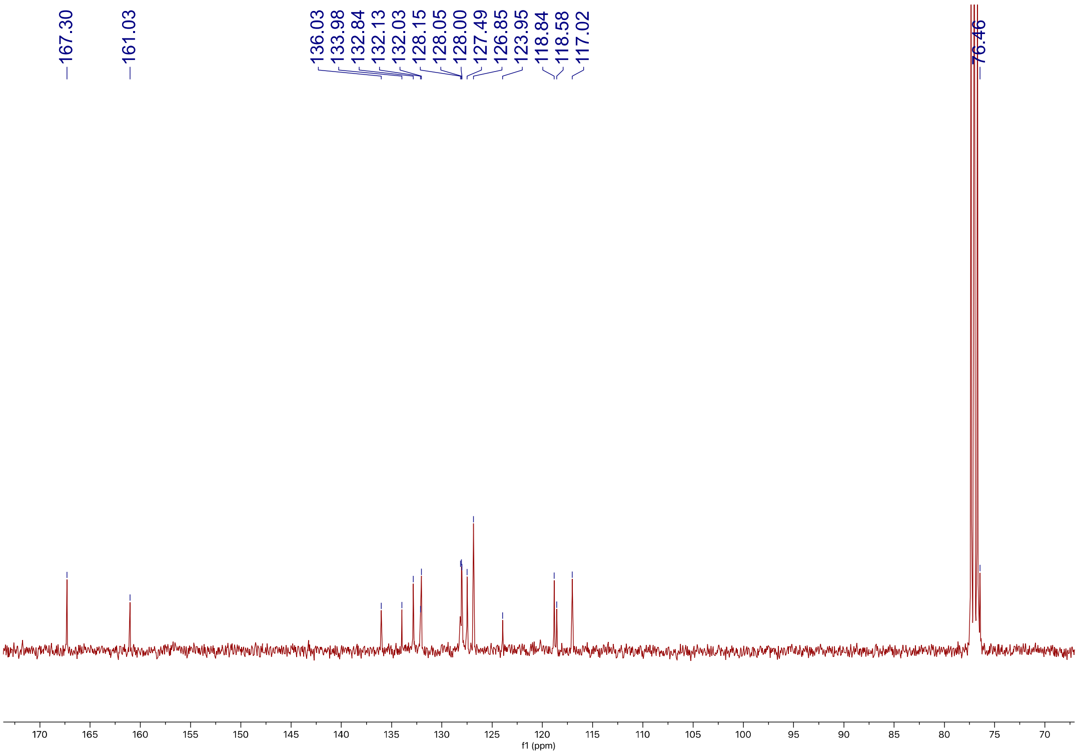

Figure S19. $^{13}C\{^{1}H\}$ NMR spectrum of **3i**.

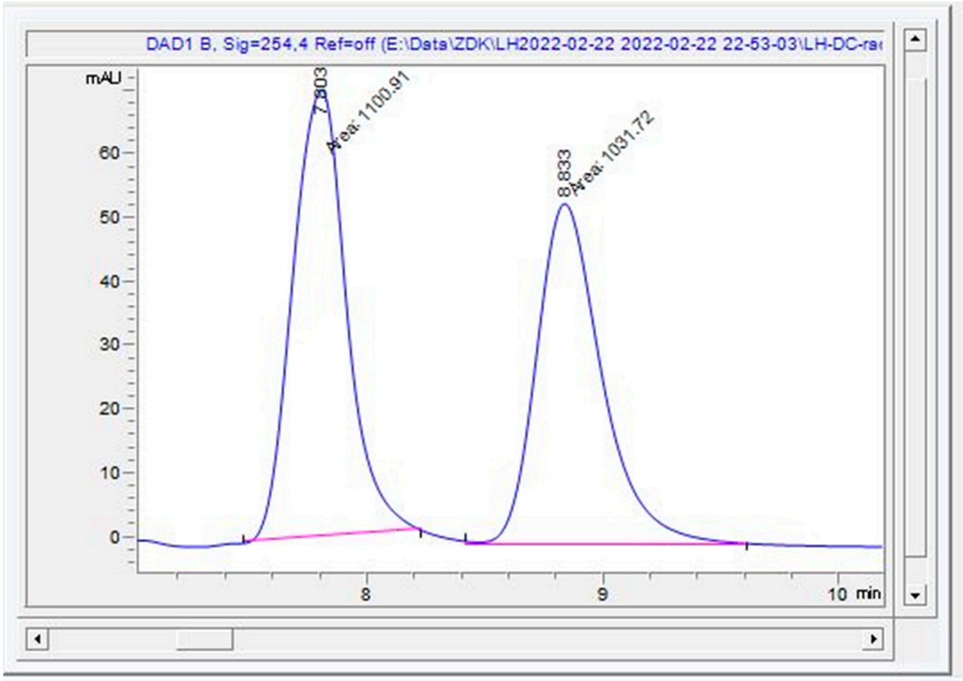

#	Time	Type	Area	Height	Width	Area%	Symmetry
1	7.803	MM	1100.9	69.7	0.2633	51.622	1.077
2	8.833	MM	1031.7	53.3	0.3228	48.378	0.725

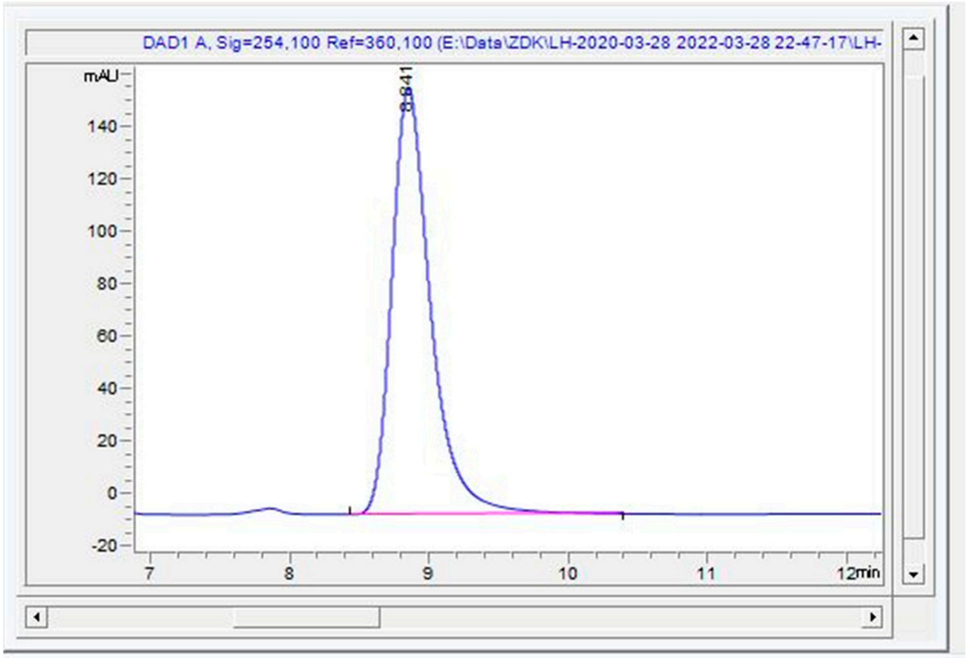

#	Time	Type	Area	Height	Width	Area%	Symmetry
1	8.841	BB	3157.4	162.1	0.2943	100.000	0.67

Figure S20. HPLC analysis of (top) racemic sample and (bottom) (*S*,*S*) sample of **3i**.
